# Supplementary material for: Self-Report versus Neuropsychological Tests for Examining Executive Functions in Youth Soccer Athletes—A Cross-Sectional Study
Source: Behav Sci (Basel). 2022 Sep 19;12(9):346. doi: 10.3390/bs12090346 (PMC9495998; doi:10.3390/bs12090346)
Supplement: Supplementary file 1 [file behavsci-12-00346-s001.zip › behavsci-1861092-supplementary.pdf]

Table S1. Correlations between performance-based EF tasks and items of the self-report of EF (BRIEF-SB).

		3-back task		cued GoNoGo task					flanker task			number-letter task					
		**MRT**	**ACC**	**ER**	**MRT**	**MRT (vert. cue)**	**MRT (hor. cue)**	**PCR**	**MRT**	**MRT (con.)**	**MRT (incon.)**	**PCR (switch)**	**PCR (no switch)**	**ACC (switch costs)**	**MRT (switch)**	**MRT (no switch)**	**MRT switch cost**
Inhibit	r (p-value)	0.107 (0.407)	-0.170 (0.179)	0.024 (0.845)	0.187 (0.127)	0.192 (0.117)	0.150 (0.223)	0.027 (0.830)	0.166 (0.179)	0.163 (0.186)	0.171 (0.167)	-0.212 (0.103)	0.022 (0.868)	-0.283* (0.028)	0.159 (0.226)	0.256* (0.048)	-0.029 (0.827)
Shift	r (p-value)	-0.073 (0.572)	-,251* (0.045)	-0.045 (0.715)	0.175 (0.154)	0.182 (0.137)	0.130 (0.289)	-0.046 (0.714)	0.098 (0.428)	0.112 (0.365)	0.073 (0.559)	-0.196 (0.133)	-0.115 (0.381)	-0.127 (0.335)	0.127 (0.332)	0.236 (0.070)	-0.053 (0.687)
Emotional Control	r (p-value)	0.002 (0.988)	-0.136 (0.285)	0.046 (0.709)	0.108 (0.380)	0.108 (0.380)	0.095 (0.441)	0.081 (0.514)	0.162 (0.191)	0.165 (0.182)	0.138 (0.266)	-0.347** (0.007)	-0.166 (0.205)	-0.262* (0.043)	0.173 (0.185)	0.170 (0.193)	0.079 (0.551)
Monitor	r (p-value)	0.014 (0.915)	-0.116 (0.363)	-0.048 (0.696)	0.371** (0.002)	0.363** (0.002)	0.350** (0.003)	0.051 (0.683)	0.058 (0.644)	0.072 (0.561)	0.031 (0.804)	-0.442** (0.000)	-0.223 (0.087)	-0.323* (0.012)	-0.068 (0.605)	-0.001 (0.993)	-0.097 (0.462)
Working Memory	r (p-value)	0.008 (0.950)	-0.148 (0.244)	-0.026 (0.832)	0.253* (0.037)	0.231 (0.058)	0.292* (0.016)	0.082 (0.508)	0.097 (0.434)	0.115 (0.355)	0.047 (0.706)	-0.215 (0.098)	-0.026 (0.846)	-0.239 (0.065)	0.088 (0.505)	0.173 (0.185)	-0.048 (0.717)
Plan/ Organize	r (p-value)	-0.114 (0.380)	-0.276* (0.028)	0.078 (0.526)	0.311** (0.010)	0.300* (0.013)	0.310* (0.010)	0.059 (0.633)	0.159 (0.199)	0.162 (0.189)	0.141 (0.254)	-0.219 (0.093)	-0.015 (0.910)	-0.254* (0.050)	0.189 (0.148)	0.347** (0.007)	-0.076 (0.563)
Organization of Materials	r (p-value)	0.002 (0.990)	-0.079 (0.536)	0.048 (0.697)	0.127 (0.301)	0.146 (0.235)	0.056 (0.651)	-0.017 (0.893)	0.089 (0.474)	0.106 (0.395)	0.040 (0.748)	-0.109 (0.406)	0.077 (0.558)	-0.211 (0.106)	0.133 (0.309)	0.163 (0.213)	0.028 (0.829)
Initiate	r (p-value)	0.109 (0.400)	-0.097 (0.447)	-0.097 (0.433)	0.141 (0.251)	0.134 (0.275)	0.146 (0.236)	0.112 (0.369)	0.029 (0.816)	0.054 (0.661)	-0.022 (0.860)	-0.274* (0.034)	-0.060 (0.647)	-0.277* (0.032)	0.267* (0.039)	0.257* (0.047)	0.126 (0.336)
Behavior Regulation Index	r (p-value)	0.018 (0.891)	-0.227 (0.071)	-0.002 (0.987)	0.251* (0.039)	0.254* (0.037)	0.211 (0.083)	0.031 (0.801)	0.166 (0.178)	0.175 (0.157)	0.145 (0.240)	-0.359** (0.005)	-0.133 (0.309)	-0.309* (0.016)	0.151 (0.249)	0.241 (0.064)	-0.024 (0.854)

Cognitive Regulation Index	r (p-value)	-0.005 (0.971)	-0.197 (0.119)	0.003 (0.981)	0.274* (0.024)	0.265* (0.029)	0.270* (0.026)	0.078 (0.530)	0.122 (0.325)	0.141 (0.254)	0.071 (0.568)	-0.260* (0.045)	-0.012 (0.927)	-0.308* (0.017)	0.209 (0.110)	0.299* (0.020)	0.000 (0.999)
Total Executive Score	r (p-value)	0.006 (0.966)	-0.228 (0.070)	0.001 (0.994)	0.288* (0.017)	0.284* (0.019)	0.267* (0.028)	0.063 (0.612)	0.154 (0.212)	0.170 (0.169)	0.113 (0.363)	-0.333** (0.009)	-0.072 (0.585)	-0.338** (0.008)	0.201 (0.124)	0.300* (0.020)	-0.012 (0.930)

Note: * = $p \leq 0.05$, **= $p \leq 0.05$
